# Cadmium oxide nanoparticles/graphene composite: synthesis, theoretical insights into reactivity and adsorption study†

**DOI:** 10.1039/d1ra04754j

**Published:** 2021-08-09

**Authors:** Dalal Z. Husein, Reda Hassanien, Mona Khamis

**Affiliations:** Chemistry Department, Faculty of Science, New Valley University El-Kharja 72511 Egypt dalal_husein@yahoo.com dalal_husein@scinv.au.edu.eg

## Abstract

Graphene-based metal oxide nanocomposites are interesting and promising kinds of nanocomposites due to their large specific area, fast kinetics, and specific affinity towards heavy metal contaminants. In this work, a facile and cost-effective route was used to synthesize CdO nanoparticles (CdO NPs) and graphene-based CdO nanocomposite (G–CdO). The prepared nanomaterials were characterized and explored for lead removal from water. Both CdO NPs and G–CdO composite exhibited excellent sorption capacity of 427 and 398 mg g^−1^, respectively, at pH 4.8 and *T* = 298 K, which was superior to individual graphene and many other adsorbents. The results indicated that the recovered nanomaterials endure 4 times recyclability retaining up to 89% lead uptake efficiency. To complement the experimental study, DFT calculations were performed to investigate the stability of the formed G–CdO composite compared to CdO NPs; the reactivity of G–CdO compared to plain graphene as well as the interaction insights between graphene and CdO clusters were studied using natural-bond-orbital (NBO), electron-localization-function (ELF) and reduced-density-gradient (RDG) analyses.

## Abstract 2

Graphene-based metal oxide nanocomposites are interesting and promising kinds of nanocomposites due to their large specific area, fast kinetics, and specific affinity towards heavy metal contaminants.

## Graphical abstract

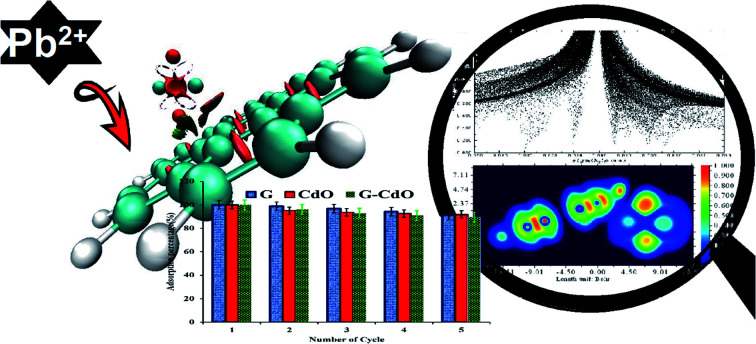

## Introduction

Pollution of water streams with heavy metals is considered a critical environmental risk all over the world owing to their highly toxic and non-biodegradable nature.^1^ Lead is considered a toxic metal even when present at trace levels.^2^ It can cause damage to brain cortex cells, the nervous system, sensory disturbance, and other symptoms.^3^ Thus, the removal of lead and other heavy metals from water streams is a vital necessity. Until now, conventional methods to remove heavy metals include coagulation,^4^ precipitation, chemical reduction,^5^ ion exchange,^6^ biological treatment,^7^ membrane separation,^8^ and filtration.^9^ Nevertheless, such techniques have drawbacks such as high cost, low purification efficiency, and difficulty in regeneration. The removal method is regarded as preferable to other methods when it has some features such as low cost, prolonged durability, and simple operation.^10^ Various materials have been used such as resin,^11^ carbon,^12^ zeolites,^13^ metal oxide,^14^ clays,^15^ biopolymers,^16^ and rice husk.^17^ However, such materials still have drawbacks because of their relatively low chemical stability and low adsorption capacity for heavy metals. Recently, graphene, single layer graphite, holds great promise for potential applications in many technological fields, especially as an adsorbent for heavy metals owing to its huge surface area, excellent thermal and conductivity due to its unique two-dimensional lattice structure.^18^ Despite the superior properties of graphene, it has a strong preference for aggregation due to strong van der Waals forces between layers of graphene sheets. Such a drawback inhibits various graphene applications but it can be eliminated by hybridizing graphene with inorganic nanomaterials, especially, with those having a higher surface to volume ratio. Thereby, a composite of graphene with inorganic nanomaterials such as metal oxides can potentially obtain a much-enhanced surface area. Therefore, it has attracted much attention in the scientific community.^19^ Various metal oxide NPs have been anchored onto graphene sheets' surfaces to act as a stabilizer against the agglomeration of these sheets due to van der Waals forces.^20^ Graphene-based metal oxide nanocomposites are an interesting and promising type of nanocomposites because of their properties that exceed the properties of their individual phases.^21^ Also, individual metal oxide nanoparticles still do not have commercial applications due to a strong tendency to aggregate as a result of van der Waals interactions. Such interactions lead to a significant reduction of selectivity and adsorption capacity with prolonged use.^22^ To overcome these shortcomings, metal oxide nanoparticles are anchored at a supporting surface as graphene, and thereby the size of metal oxide NPs is stabilized on the matrix of the nanocomposite. As a result, the total surface area increases and the adsorption capacity will improve. Moreover, incorporating graphene with metal oxide NPs will prevent the loss of metal oxide nanoparticles due to the stabilization or anchoring of the nanoparticles onto the surface of graphene sheets,^23^ which makes the application of graphene-based metal oxide nanocomposites becoming increasingly popular in environmental applications.^24^ In literature, graphene has been hybridized with metal oxides such as Ni/Fe_2_O_3_,^25^ MnO_2_,^26^ Fe_3_O_4_,^27^ SiO_2_,^28^ ZnO^29^ to formulate adsorbents for heavy metal removal. Our group designed a facile, cheap, and eco-friendly graphene-based CdO nanocomposites *via* a green approach.^30^ Green route synthesis has many great advantages to make it outstanding among all preparation routes to remove heavy metals from aquatic medium.^31^ Although there are many experimental studies have investigated the potential of graphene-based metal oxides for application as efficient adsorbents for various pollutants, experimental information is not enough to explain the whole picture of the interaction between graphene and metal oxides. In this context, computational chemistry can provide many detailed insights into the interaction between metal oxides and graphene as well as the reactivity and stability of the composite compared to individual graphene. Density functional theory (DFT) as a tool can provide deeper insights to better understand interactions and reactivity in graphene composites.^32^

In this study, a graphene–CdO composite was prepared and characterized using various techniques and its adsorption efficiency towards lead removal from the water was explored. The impact of various sorption factors, such as the initial concentration of Pb(ii), reaction time, temperature, pH, and solid dose on Pb(ii) uptake, was studied using batch techniques. The thermodynamic, kinetic, and isotherm studies were performed to elucidate the mechanism of the sorption process. Regeneration efficiency and recyclability were studied as well. The interaction between CdO and graphene will be discussed by DFT calculations as well as the reactivity of formed nanocomposite compared to single graphene towards the removal of lead ions from water.

## Materials and methods

### Chemicals

Na_2_SO_4_, 99% and Cd(CH_3_COO)_2_·2H_2_O, 99.5%, ethanol, HCl, HNO_3_, NaOH and Pb(CH_3_COO)_2_ were purchased from LOBA CHEMIE. Hp pencil cores were used as a source of graphite. All chemical reagents were pure and used without purification.

### Synthesis of nanoadsorbents

#### Graphene

Graphene was prepared by the electrochemical exfoliation method.^30^ In brief, two graphite pencil cores were installed as cathode and anode electrodes in an electrochemical cell and 1 M Na_2_SO_4_ solution was used as a conductive electrolyte. The exfoliation process was applied under 12 volts. The obtained graphene sheets that precipitated as black debris, were washed with de-ionized water, and then sonicated for about 30 min for complete exfoliation. Exfoliated graphene sheets were washed and dried at 60 °C for about 6 h.

#### Preparation of onion extract

Red onions (100 g) were sliced, and dipped in aqueous ethanol (50 : 50 v/v), for 2.5 days. Then the onion residue was removed and the solution was stored at 20 °C for further use.

#### Plant-mediated-synthesis of CdO nanoparticles (CdO NPs)

50 mM of aqueous Cd(CH_3_COO)_2_·2H_2_O solution was added to about 300 mL extract under stirring. A yellow precipitate was formed immediately. The reaction solution was aged for 24 h at room temperature and pH 5.5 and then centrifuged and washed several times with deionized water and then ethanol. Afterward, the obtained material was dried at 60 °C then calcined at 400 °C for 2 h.^30^

#### Synthesis of graphene-based CdO nanocomposite (G–CdO)

About 0.3 g exfoliated graphene was diffused in 300 mL onion extract at room temperature. Then, a solution of 50 mM of cadmium acetate was mixed with the graphene mixture and stirred at room temperature for 30 min. The precipitate was centrifugated, washed, dried, and calcined as performed during CdO-NPs preparation.

### Characterization

Various instruments were used to characterize the formed nanomaterials, G, CdO and G–CdO. FT-IR spectrophotometer (Thermo Fisher – Nicolet iS10), UV-Vis spectrophotometer (JASCO 530, Japan), X-ray diffractometer (Philips – PW1710), transmission electron microscopy – TEM; (JEOL – JEM-100 CXII), scanning electron microscopy – SEM; (QUANTAFEG 250 with energy-dispersive X-ray spectroscopy – EDX attachment) and Raman spectrometer (WITEC Alpha300 laser) were utilized.

### Adsorption experiments

All tests were performed at 29 °C using 25 mL of lead ion solution in a batch mode. The effects of pH (1.55–7.02), nanoadsorbent dose (0.015–0.03 g), and lead initial concentration (25–1500 mg) on the sorption behavior were studied. The kinetic study was performed by adding 0.025 g of nanoadsorbent to Pb(ii) solutions with initial concentrations of 550 mg L^−1^ at pH 4.8 and the residual lead ions concentration was measured (at time = 0–24 h) using an ICP-OES (inductively coupled plasma optical emission spectrometry, Thermo Co., model ICAP6500 Duo, England). For the thermodynamic studies, 0.025 g of nanoadsorbent was added to 25 mL of 550 mg L^−1^ Pb(ii) solution at pH 4.8 for 24 h at various temperatures (25–37 °C). The pH during the sorption experiments was regulated by dropwise addition of 0.1 M HNO_3_ or 0.1 M NaOH.

### Regeneration performance

Specific amounts of lead-loaded nanomaterials were eluted with dilute hydrochloric acid solution and then washed until the pH was neutral. Hence, the spent materials were dried at 75 °C to constant weight, and then re-loaded with Pb(ii) ions to investigate the service life of nanomaterials and % removal.

### Adsorption calculations

The sorption capability (*q*_*t*_; mg g^−1^) and the percent removal or uptake (*R*%) of lead ions at equilibrium were calculated using eqn (1) and (2),

1 *q*_*t*_ = (*C*_0_ − *C*_e_)*V*/m

2 *R*% = [(*C*_0_ − *C*_e_)/*C*_0_] × 100

*C*_0_ and *C*_e_ (mg L^−1^) are the lead concentrations in the liquid phase before and after adsorption, respectively. *V* (L), *t* (min), and *m* (g) are the volume of the liquid phase, time, and mass of the dry nanoadsorbent used, respectively.

The results of adsorption kinetic experiments were studied by four models: the pseudo-first- and second-order, intraparticle, and Elovich models. Pseudo-first-order, eqn (3) presumes that the sorption rate depends on the removal capacity.^33^

3 log(*q*_e_ − *q*_*t*_) = log *q*_e_ − (*K*_1_/2.303)*t*

*q*_e_ and *q*_*t*_ are the Pb(ii) concentration of sorbed by nanomaterial (mg g^−1^) at equilibrium phase and predetermined time interval *t*, respectively. *K*_1_ is the pseudo-first-order rate constant (min^−1^).

The pseudo-second-order kinetic model, eqn (4), indicates that the adsorption process is controlled by the chemical interaction mechanism, including sharing between the contaminant and the adsorbent material or valency forces through the electron transfer.^34^

4 (*t*/*q*_*t*_) = 1/(*K*_2_*q*_e_^2^) + (1/*q*_e_)*t*

where *K*_2_ (mg g^−1^ min^−1^) is the rate constant of the pseudo-second-order. Another model is Elovich, which is symbolized as eqn (5):^35^

5 *q*_*t*_ = (1/*β*)(ln *αβ*) + (1/*β*)ln(*t*)

where *α* is the initial adsorption rate parameter of lead ions (mg g^−1^ min^−1^) while *β* (g mg^−1^) is Elovich constant correlates activation energy and the surface coverage included in chemisorptions.

The intraparticle diffusion equation is expressed as eqn (6) to explore the intraparticle diffusion mechanism:^36^

6 *q*_*t*_ = *C* + *K*_int_(*t*)^1/2^

where *K*_int_ (mg g^−1^ min^−1/2^) is the rate constant of the model. Values of *C* describes the boundary thickness. This means the larger is the intercept (*C*), the greater is the effect of the boundary layer in the solution. The values of *K*_int_ (slope) reflect the sorption rate of lead ions onto the nanomaterial surface.

After completion of adsorption isotherm tests, four models, Langmuir, Temkin, Freundlich, and Dubinin–Redushkevich (D–R) were applied to depict all isothermal features of G–CdO nanoadsorbent. The four models can be written (linear form) as in eqn (7)–(10). Langmuir's model describes the adsorption process as a chemical interaction, suggesting that the sorptive active sites of the nanomaterial surface are uniform and one layer of the adsorbate is formed, eqn (7).^37^

7 *C*_e_/*q*_e_ = (1/*q*_L_*K*_L_) + (1/*q*_L_)*C*_e_

where *q*_L_ is the maximum sorption capability (mg g^−1^) of nanomaterial adsorbents and *K*_L_ is a constant (L mg^−1^) describing sorption affinity for the nanoadsorbent. The basic characteristic of that model could be described in terms of a dimensionless factor, *R*_L_, which is expressed as (8):

8 *R*_L_ = 1/(1 + *K*_L_*C*_max_)

and *C*_max_ is the highest Pb(ii) concentration.

Freundlich describes a heterogeneous surface of nanoadsorbent^38^ and defined as (9):

9 log *q*_e_ = log *K*_F_ + (1/*n*)log *C*_e_

The *K*_F_ parameter, (mg g^−1^) (L mg^−1^)^1/*n*^ is a constant corresponding to the Pb(ii) multilayer sorption capacity. The constant *n* is defined as the heterogeneity factor.

Temkin isotherm model depicts that the heat of Pb(ii) sorption is gradually reduced linearly with surface interaction^39^ and the linear form is better for the sorption process rather than the logarithmic, eqn (10):

10 *q*_e_ = *B* ln *A* + *B* ln *C*_e_

11 *B* = *RT*/*b*

where *A* (L g^−1^) is the Temkin isotherm equilibrium binding constant while *B* corresponds to equilibrium adsorption heat. The gas constant is *R* (J mol^−1^ K^−1^) and the absolute temperature is *T*.

The D–R isotherm was approached from the following eqn (12):^40^

12 ln *q*_e_ = ln *q*_m_ − *βε*^2^

*β* is a D–R parameter that correlates free energy; *E*; (kJ mol^−2^ where *E* = (−2*β*)^−1/2^), while *ε* (J mmol^−1^) is Polanyi potential defined as eqn (13):

13 *ε* = *RT*(1 + 1/*C*_e_)

The data of the thermodynamic experiments were analyzed to get the change in free energy (Δ*G*^0^, kJ mol^−1^), standard entropy (Δ*S*^0^ J K^−1^ mol^−1^), and standard enthalpy (Δ*H*^0^, kJ mol^−1^) as:14
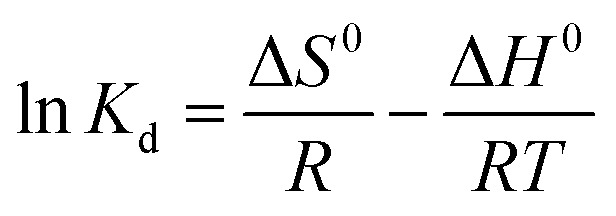

15 Δ*G*^0^ = −*RT* ln *K*_d_

*K*_d_ represents linear sorption coefficient, defined as a ratio between the surface concentration of adsorbed Pb(ii) and its concentration in the solution at equilibrium.

### Computational methods

All computational calculations were performed on a personal computer and Gaussian 09W program package. The B3LYP^41^ functional was adopted with LANL2DZ as a basis set for all performed calculations. All structures and Gaussian output files were generated and visualized by using Gaussian view 05 program. The geometry optimization of G and G–CdO molecules was performed using DFT schemes. No imaginary frequencies were detected, which ensured the minimum potential energy surface of the proposed structures. The global descriptors of chemical reactivity for G and G–CdO were examined. The HOMO and LUMO energy values; highest occupied and lowest unoccupied molecular orbitals, respectively, are the starting point to get all global reactivity descriptors. According to Koopmans' theory, the ionization potential (*I*) and affinity (*A*) are written as:

16 *I* = −HOMO, *A* = −LUMO

Global potential (*μ*), global hardness (*η*), electronegativity (*χ*), electrophilicity index (*ω*), index of softness (*σ*), maximal charge acceptance (Δ*N*_max_) and energy change (Δ*E*) are stated as:

17 *μ* = (*I* + *A*)/2

18 *η* = (*I* − *A*)

19 *χ* = −*η*

20 *ω* = *μ*^2^/(2*η*)

21 *σ* = 1/*η*

22 Δ*N* = −(*μ*/*η*)

23 Δ*E* = −*ω*

The interactions between graphene and CdO in the formed composite were explored using natural-bond-orbital (NBO) analysis, electron-localization-function (ELF) and reduced-density-gradient (RDG) analyses. Perturbation delocalization or stabilization energies of second-order (*E*^2^) were calculated using the NBO 5.9 software (in Gaussian) at level DFT/B3LYP/LANL2DZ of the theory to determine importantly donor–acceptor interactions in the G–CdO complex. Multiwfn, Gauss Sum and VMD programs were also used at the same level of theory.

## Results and discussions

### Characterization of prepared adsorbents

**Fig. 1:**
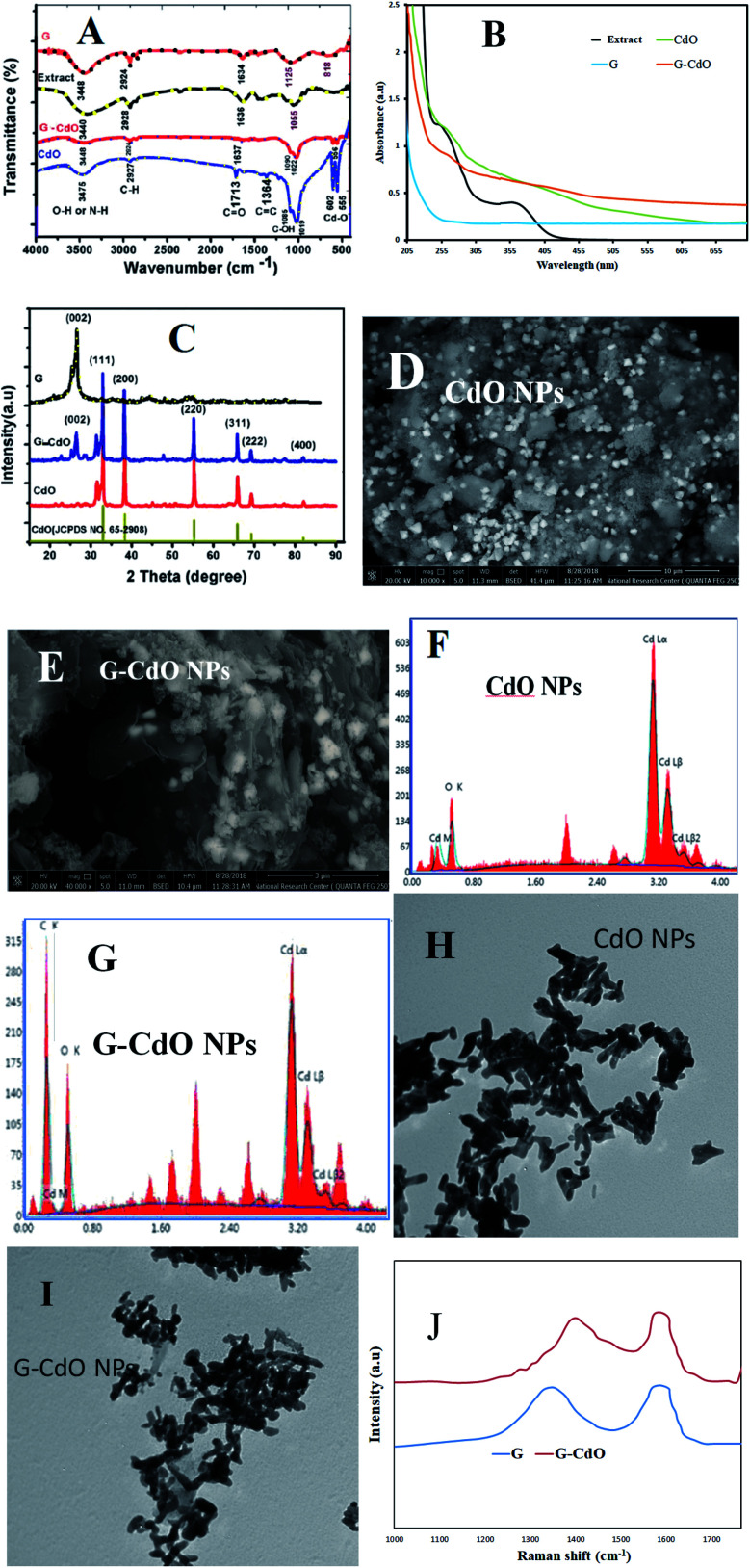
Characterization of prepared adsorbents: (A) FTIR spectra; (B) UV-Vis absorption spectra for onion extract and prepared adsorbents; (C) XRD analysis; (D and E) SEM images; (F and G) EDX analysis; (H and I) TEM analysis and (J) Raman analysis.

The FT-IR spectrum of graphene, Fig. 1A, showed various bands corresponding to stretching groups O–H (at 3448 cm^−1^), C–H (at 2924 cm^−1^), C=C (at 1634 cm^−1^), and C–O (1090 cm^−1^). Onion extract spectrum showed peaks attributed to O–H and N–H at 3440 cm^−1^, C–H asymmetric at 2928 cm^−1^, carbonyl vibration at 1636 cm^−1^, C=C aromatic at 1425 cm^−1^, and C–O at 1055 cm^−1^. These data reflected the complex nature of the extract derived from polyphenols, carbohydrates, proteins, and lipids. For the prepared nanomaterials spectra, a similarity is displayed with slight changes in intensities or positions, which depict the presence of onion organic species as capping and complexing agents. The Cd–O vibrational stretching appears at 603 and 556 cm^−1^ in composite and single cadmium oxide spectra, which affirms the formation of nanomaterials.

UV-vis absorptions, Fig. 1B, of the graphene, showed an aromatic π–π* transition at 265 nm, while the extract showed two absorption bands assigned to the proteins (at 253 and 350 nm). An interband transition was seen in the spectrum of CdO NPs at 260 nm. The spectrum of G–CdO showed such interband transition at 263 nm with a shoulder at 232 nm, characteristic of graphene. Around 398 nm, both spectra, of single CdO and its composite, showed a band that refers to the quantum confinement of these nanomaterials.

The XRD analysis, shown in Fig. 1C, indicated an intense diffraction peaks ascribed to (111) at 2*θ* = 33.01, (200) at 2*θ* = 38.33, (220) at 2*θ* = 55.34, (311) at 2*θ* = 65.8, (222) at 2*θ* = 69.31 and (400) at 2*θ* = 82.03 the cubic structure of CdO (JCPDS 65-2908). The XRD analysis of graphene showed an intense peak at 26.56° related to the (002) plane that confirms the exfoliation of graphite into graphene layers. The XRD analysis of G–CdO is similar to both hybrid CdO NPs and graphene reflect the anchoring of CdO nanoparticles onto the nanosheets of graphene. The mean particle sizes calculated from the Scherrer equation were 21.94 and 26.09 nm for CdO and G–CdO, respectively. The peak sharpness of the prepared nanomaterials indicates their high crystalline nature. Such finding is harmonious with the SEM analysis, Fig. 1D and E, which showed highly crystalline polygonal (mainly cubic and octahedrons) particles that affirm the preparation of CdO and affirm its anchoring onto graphene nanosheets. EDX results of CdO NPs and G–CdO are shown in Fig. 1F and G, respectively, and they indicated the formation of highly pure CdO and hybrid G–CdO nanomaterials.

The TEM analysis of CdO NPs and G–CdO are shown in Fig. 1H and I, respectively, it indicated disordered chain mixtures with accumulation in specific places, and the surface of graphene sheets was decorated by spherical CdO NPs. The particle sizes were found to be in the range 18–27 and 19–38 nm for nano CdO and G–CdO, respectively. These results are similar to the average sizes determined by the Scherrer equation.

To provide additional evidence on G–CdO formation, Raman analysis was carried out for the prepared graphene and G–CdO nanocomposite. Raman spectroscopy is an effective tool to distinguish ordered and disordered carbonaceous materials. The spectrum of graphene shown in Fig. 1J showed two broad peaks at 1348 cm^−1^ (D-band) and 1585 cm^−1^ (G-band), attributed to a primary in-plane vibration of *k*-point phonons belonging to A_1g_ symmetry and first-order in-plane stretching vibration belongs to E_2g_ phonon from sp^2^ carbon rings, respectively.^42^ The Raman spectrum of G–CdO shows a clear redshift for both D and G peaks by 10 and 8 cm^−1^, respectively, which confirm the anchoring of CdO on the graphene surface. The intensity ratio (*I*_G_/*I*_D_) usually measures the degree of defects in the graphene system.^43^ The calculated *I*_G_/*I*_D_ ratio was increased from 1.03 (G) to 1.08 (G–CdO) implies a high degree of defects. This finding adds to the proof that the G–CdO composite was formed.

### Adsorption studies

#### Effect of pH

**Fig. 2:**
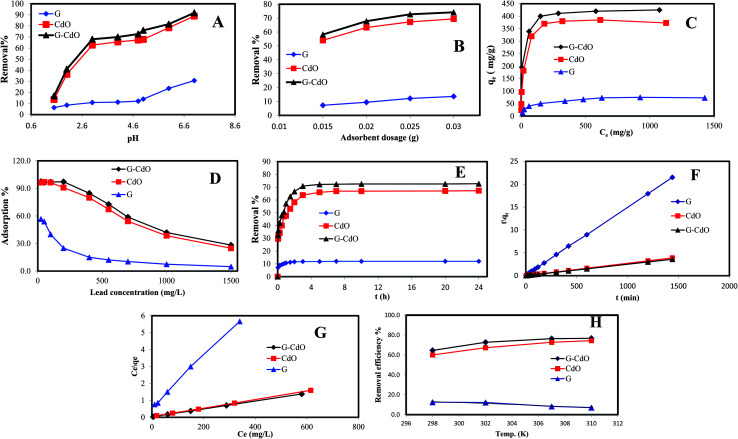
Removal of Pb(ii) ions by graphene, CdO NPs and G–CdO nanocomposite: (A) effect of initial solution pH, (B) effect of adsorbent dose, (C and D) effect of Pb(ii) concentration, (E) effect of time, (F) pseudo-second order kinetic model, (G) Langmuir isotherm, and (H) effect of temperature.

Fig. 2A demonstrates the pH-based adsorption capacity for Pb(ii) ions for 24 h contact time at 550 mg L^−1^ as an initial lead concentration and solid/liquid ratio of 1g L^−1^. The highest adsorption capacity was noted for the G–CdO nanocomposite compared to CdO NPs and graphene, which may be attributed to the synergistic effects of CdO NPs and graphene. The adsorption capacity of the graphene was found to be the lowest in an acidic medium and increased with increasing solution pH. As seen in Fig. 2A, the lower adsorption efficiency in acidic solutions may be assigned to the competitive presence of proton ions. As the solution pH increased, the proton ions concentration decreased, leading to enhance adsorption efficiency. When pH was raised from 1.5 to 5.0, the adsorption capacity of nanoadsorbents was also raised due to electrostatic interaction between Pb(ii) and graphene rings. Besides, the chelate formation of Pb(ii) with CdO that decorated the graphene surface could have been involved. However, as moving from acidic to a neutral solution, the interaction with lead ions is favorable, resulting in the enhancement of the removal capacity until Pb(ii) ions tend to hydrolyze.^44^ Owing to the subsequent hydrolysis and precipitation of lead ions in basic solution, the pH of the medium was set to 4.8 for all experimental studies to avoid Pb(ii) precipitation.

#### Effect of nanoadsorbent dose

The amount of nanoadsorbent affects the rate of Pb^2+^ adsorption. Hence, different doses of nanoadsorbents were used for the lead removal. As shown in Fig. 2B, a rise in the lead removal capacity can be noted with the mass ratio of solid/liquid in the initial period, followed by a very slow increase at the amount of 0.025 g. This could be related to effective active sites on the nanomaterial surface remarkably increased as the nanoadsorbent dose increases, leading to the rapid uptake of lead ions from the sorption solution. Nevertheless, when the nanomaterial dose continued to increase, the uptake efficiency of lead ions will reach the equilibrium stage. It may be considered a saturation point. Any increase in the solid dose after this saturation point leads only to an increase in the thickness of the Pb(ii) adsorbed layer at the adsorbent surface. Again, the removal efficiency was higher for G–CdO nanocomposite followed by CdO NPs.

#### Effect of lead ion concentration

The influence of lead concentration on the removal capacity of prepared adsorbents is displayed in Fig. 2C and D. The maximum sorption capacity towards lead ions is an important feature of the nanomaterial as an adsorbent. As seen in Fig. 2C, the lead uptake capacity increases dramatically with an increase in lead concentration. After exceeding 550 mg L^−1^, the sorption capacities almost remained constant. This is possibly attributed to the lack of adequate active sites to accommodate additional Pb(ii) ions. The maximum lead adsorption capacities for G–CdO, CdO NPs, and G are 400, 370, and 67 mg g^−1^, respectively.

The lead removal% for three prepared nanoadsorbents, shown in Fig. 2D, rose initially then decreased after Pb(ii) concentration reached 200, 100, and 50 mg g^−1^ for G–CdO, CdO and G, respectively. During the increase in initial lead concentration, the surface areas of the three nanoadsorbents available for the removal are insufficient, hence, the rate of lead removal decreases with increasing lead concentration. At lead concentration = 200 mg L^−1^ for G–CdO, 100 mg L^−1^ for CdO NPs and 50 mg L^−1^ for graphene, equilibrium was attained since all the adsorption sites on the surface of the nanomaterials were bonded by lead ions. As illustrated in Fig. 2D, the uptake efficiency of the hybrid G–CdO nanocomposite was the highest.

#### Effect of contact time

Fig. 2E shows the impact of time within the range of 0–24 h. At 0–3.7 h, the uptake of lead ions increased sharply. Afterward, the percent removal remained steady and, in some cases, decreased at higher times. Such effect may be assigned to the desorption of the lead ions from the nanoadsorbent surfaces during a long time of contact with the aqueous medium. The lead removal% increase in the order G–CdO > CdO NPs > G and in 3.7 h reached 72.7, 67.3, and 12.2%, respectively. This illustrated that the nanocomposite material effectively had a higher selectivity than the individual components, graphene, and CdO nanoparticles. The better uptake rate of CdO NP and G–CdO nanocomposite than graphene may be attributed to their huge surface area and higher amounts of adsorptive sites on their surface that cumulated organic functional groups derived from onion extract. For graphene, the lead removal capacity was limited over time during the adsorption experiments, which may be restricted by the cover of active sites after aggregation. The anchoring of cadmium oxide nanoparticles onto the surface of graphene nanosheets provided a higher adsorption capacity of the hybrid nanocomposite than individual graphene.

### Kinetic studies

Kinetics parameters were calculated and are shown in Table 1 and Fig. 2F. Based on the correlation coefficient values, *R*^2^, the kinetics data of lead adsorption onto G, CdO and G–CdO were better supported by the pseudo-second-order model than by other models, as shown in Table 1. The correlation coefficient values for the three adsorbents were extremely high with good linearity (*R*^2^ > 0.990). Deviation from linearity for the rest models reflects the lack of fitting of these models to the experimental kinetic data.

**Table 1:** Kinetic parameters for lead removal onto G–CdO, CdO and G

Adsorbents	Kinetics models			
	Pseudo-first-order	Pseudo-second-order	Elovich	Intraparticle diffusion
G–CdO	*q*_e_ = 69.2	*q*_e_ = 403.4	*α* = 1.3 × 10^3^	*K*_int_ = 5.48
	*K*_1_ = 0.0039	*K*_2_ = 0.00023	*β* = 0.025	*C* = 248
	*R* ^2^ = 0.6851	*R* ^2^ = 0.9999	*R* ^2^ = 0.9199	*R* ^2^ = 0.6266
CdO	*q*_e_ = 82.9	*q*_e_ = 374.3	*α* = 0.62 × 10^3^	*K*_int_ = 5.58
	*K*_1_ = 0.0035	*K*_2_ = 0.00018	*β* = 0.025	*C* = 210.5
	*R* ^2^ = 0.7652	*R* ^2^ = 0.9998	*R* ^2^ = 0.9295	*R* ^2^ = 0.7029
G	*q*_e_ = 8.6	*q*_e_ = 67.3	*α* = 9.5 × 10^3^	*K*_int_ = 0.57
	*K*_1_ = 0.0021	*K*_2_ = 0.0021	*β* = 0.208	*C* = 50
	*R* ^2^ = 0.5491	*R* ^2^ = 0.9999	*R* ^2^ = 0.9076	*R* ^2^ = 0.5089

In general, the pseudo-second-order model was employed to represent chemisorption, with ionic force relating to the electron sharing or exchange between the lead cations and nanomaterial as covalent interaction, and ion exchange. As a result, the chemisorption process governed the rate of lead removal by G, CdO and G–CdO. Furthermore, it was found that the experimental values of adsorption capacities (400, 370, and 67 mg g^−1^ for G–CdO, CdO NPs and G, respectively) were very close to the calculated values (403.4, 374.3, and 67.3 mg g^−1^ for G–CdO, CdO NPs and G, respectively). This finding indicates that the pseudo-second-order model can accurately express lead adsorption onto prepared nanomaterials.

### Isotherms

Dubinin–Redushkevich, Freundlich, Langmuir, and Temkin sorption isotherms were utilized to clarify the lead removal mechanism. Calculated results from various adsorption isotherms, Table 2, revealed that the process suited very well for the Langmuir model with *R*^2^ values of 0.9998, 0.9998, and 0.9981 for G–CdO, CdO NPs, and G, respectively, Fig. 2G. The Langmuir isotherm is premised on the idea that adsorption occurs at homogenous active sites and that after monolayer adsorption, the adsorbent's surface is saturated. The model further revealed that adsorption feasibility is determined by the dimensionless separation factor (*R*_L_). Adsorption is either favorable (0 < *R*_L_ < 1), linear (*R*_L_ = 1), unfavorable (*R*_L_ > 1), or irreversible (*R*_L_ = 0) based on the *R*_L_ value. The better-fitting Langmuir isotherm model suggested the homogenous adsorption of lead cations with all adsorption active sites of equal affinity. The separation factor, *R*_L_, values determined from the Langmuir model for lead removal were in the range of 0.095–0.018 suggesting that the removal process was favorable. The Langmuir monolayer adsorption capacities for lead removal systems increased from 79 mg g^−1^ (for G) to 427 mg g^−1^ (for G–CdO), demonstrating that the modified G–CdO is capable of removing lead from the aqueous solution.

**Table 2:** Isotherm parameters for lead removal onto G–CdO, CdO and G

Adsorbents	Isotherm models			
	Langmuir isotherm	Freundlich	Temkin	Dubinin–Radushkevich
G–CdO	*q*_m_ = 427	*K*_F_ = 153	*A* = 10.4	*q*_m_ = 392
	*K*_L_ = 0.092	*n* = 5.8	*B* = 51.07	*E* = 0.349
	*R*_L_ = 0.0184		*b*_T_ = 0.049	
	*R* ^2^ = 0.9998	*R* ^2^ = 0.9356	*R* ^2^ = 0.9591	*R* ^2^ = 0.9361
CdO	*q*_m_ = 398	*K*_F_ = 108	*A* = 1.76	*q*_m_ = 367.6
	*K*_L_ = 0.055	*n* = 4.6	*B* = 59.5	*E* = 0.111
	*R*_L_ = 0.030		*b*_T_ = 0.042	
	*R* ^2^ = 0.9998	*R* ^2^ = 0.8623	*R* ^2^ = 0.8994	*R* ^2^ = 0.9653
G	*q*_m_ = 79	*K*_F_ = 8.1	*A* = 13.6	*q*_m_ = 56.15
	*K*_L_ = 0.015	*n* = 2.9	*B* = 0.29	*E* = 0.126
	*R*_L_ = 0.095		*b*_T_ = 182.6	
	*R* ^2^ = 0.9981	*R* ^2^ = 0.9225	*R* ^2^ = 0.9936	*R* ^2^ = 0.8851

From Table 2, the values of constant *n*, for the adsorption of lead are higher than 1, indicating that the uptake process was favorable. Therefore, the uptake of lead ions by G–CdO, Cd NPs and graphene surface proceed with some heterogeneous inactive sites.

According to the calculated Temkin parameters *B* and *A*, Table 2, values were found to be higher in the case of G–CdO and CdO NPs (51.07 L g^−1^, 10.4 J mol^−1^ and 59.5 L g^−1^ and, 1.76 J mol^−1^, respectively) than graphene (13.6 L g^−1^, 0.29 J mol^−1^) indicating that the heat of sorption refers to a physical process.

Although the Dubinin–Redushkevich model does not fit the sorption process, the values of *E* in Table 2 confirmed the physical process. The order of isotherms is: Langmuir > Freundlich > Temkin > Dubinin–Redushkevich model.

### Adsorption performance evaluation

A comparison of the sorption performance of prepared nanoadsorbents with other reported adsorbents for lead is shown in Table 3. The synthesized CdO nanoparticles and hybrid G–CdO are considered to have the outstanding capability to adsorb Pb(ii) cations compared to various nanoadsorbents (SiO_2_/graphene,^28^ TiO_2_/graphene oxi,^45^ reduced graphene oxide/FeNPs,^46^ PDA@reduced graphene oxide/Fe_3_O_4_,^47^ Fe_3_O_4_/chitosan/graphene oxide^48^ to remove lead from water, Table 3). The uptake capacities of both CdO and G–CdO composites were 5.3 and 5.5 times, respectively, greater than the removal capacity of pristine graphene. The excellent adsorption capacity of the synthesized nanomaterials, in this study, was assigned to a huge specific surface area and dispersion of CdO onto graphene sheets. The result revealed that a relatively lower dosage of G–Cd was needed for the sorption process. The facile and green synthetic route and excellent adsorption performance may lead to broad application prospects for G–CdO nanocomposite.

**Table 3:** Comparison of removal capacity of lead ions by graphene or graphene oxide-based metal oxide nanocomposites from aqueous solution

Graphene or graphene oxide composite	Adsorption capacity (mg g^−1^)	References
TiO_2_/graphene oxide	2.7	45
Reduced graphene oxide/FeNPs	6	46
PDA@reduced graphene oxide/Fe_3_O_4_	35.2	47
Fe_3_O_4_/chitosan/graphene oxide	76.94 mg g^−1^	48
SiO_2_/graphene	113.6	28
G–CdO	427	This study
CdO NPs	398	This study
Graphene	79	This study

### Thermodynamic studies

To investigate the sorption process of Pb(ii) in-depth, typical thermodynamic parameters were determined. For pristine graphene, the calculated parameters in Fig. 2H and Table 4, indicated an exothermic (Δ*H*^0^ < 0) reaction.^1^ The negative Δ*S*^0^ suggests a decrease in the randomness in the graphene/Pb(ii) system during the removal process. Positive values of Δ*G*^0^ under various temperatures denoted that the reaction was a nonspontaneous process.^1^ The same finding was reported for the adsorption of Pb(ii) by reduced graphene oxide.^49^ From Table 4, it is clear that the Δ*G*^0^ value for both CdO and G–CdO adsorbents are negative indicating that the two reactions are a spontaneous and thermodynamically feasible process. As illustrated in Fig. 2H, a higher temperature enhances the adsorption reaction, and the values of Δ*H*^0^ are positive.

**Table 4:** Thermodynamic parameters for lead removal onto G–CdO, CdO and G

Adsorbents	Temperature (K)	*K*_d_ (L mg^−1^)	Δ*G*^0^ (kJ mol^−1^)	Δ*H*^0^ (kJ mol^−1^)	Δ*S*^0^ (J mol^−1^ K^−1^)
G–CdO	298	1.82	−1.48	37.59	131.8
	302	2.67	−2.46		
	307	3.23	−2.99		
	310	3.3	−3.07		
CdO	298	1.5	−1.0	42.35	145.38
	302	2.06	−1.81		
	307	2.67	−2.5		
	310	2.9	−2.74		
G	298	0.14	4.81	−43.36	−161.02
	302	0.14	4.95		
	307	0.09	6.11		
	310	0.08	6.63		

In a similar work, Ren *et al.*^50^ noticed an increase in graphene/MnO_2_ capacity from 46 to 60 mg g^−1^ when the reaction temperature was raised from 298 K to 318 K. In this study, at 310 K the adsorption capacity of both CdO NPs and G–CdO nanocomposite is observed to be 1.2 times higher than for those recorded at 298 K. The Δ*S*^0^ of two adsorbents are positive, indicating that the randomness at the adsorbent/Pb(ii) solution interface increased as the process progressed. The values of Δ*H*^0^ were ranged from 37 to 43 kJ mol^−1^, illustrating a physisorption reaction.

### DFT calculations

**Fig. 3:**
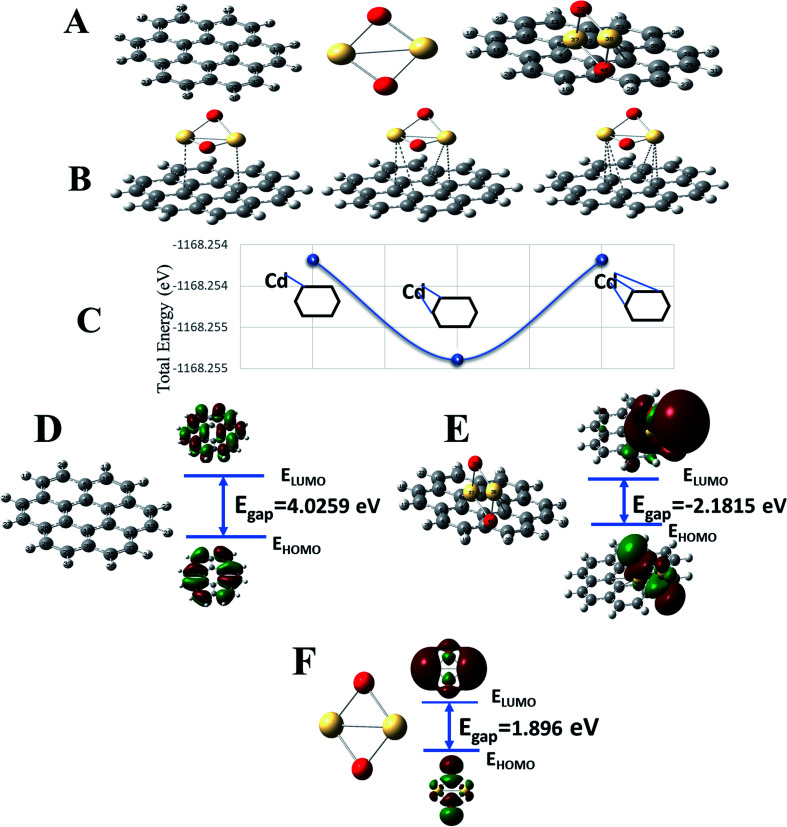
Constructed models for G, CdO and G–CdO (A), possible binding in G–CdO complex (B) and total energy for the three suggested binding structures (C), optimized structures, frontier molecular orbitals (HOMO–LUMO) and related transition energy of G (D), G–CdO (E) and CdO (F) estimated at the DFT, B3LYP with LANL2DZ basis set.

To complement experimental results, DFT calculations were performed to respond to three questions: (1) the stability of formed G–CdO nanocomposite compared to CdO, (2) the reactivity of the composite compared to plain graphene and CdO, and (3) the interaction insights between G and CdO clusters in the prepared composite. For easy calculations, a dimer of CdO cluster was considered and a graphene sheet was built as a hexagonal pattern with 7 benzene rings (24-C atoms). CdO was added to the graphene sheet at the central benzene ring. Fig. 3A illustrates the constructed models of graphene, CdO and graphene functionalized with CdO with the atom numbering scheme. The schematic possible binding of CdO into the central benzene ring is demonstrated in Fig. 3B. Each cadmium atom can be bound to either one or two or three carbon atoms in the central benzene ring, as illustrated in Fig. 3B. The calculations of total energy, Fig. 3C, showed that energies of the double-bonded Cd-rings at C4 and C8 and C10 and 7 are better than the single and triple bonded Cd-rings. Therefore, the double-bonded system was adopted to perform the remaining calculations.

### Chemical stability and global reactivity of the nanocomposite

To characterize the chemical stability and reactivity of the G–CdO complex, the energies of electronic parameters; HOMO, LUMO and their electronic gaps, *E*_gap_ for CdO, G, and G–CdO systems are demonstrated in Fig. 3D–F, and all calculated parameters are listed in Table 5. The obvious changes in energies of HOMO and LUMO have been induced in the G–CdO complex, which can be attributed to the deposition of CdO nanoparticles into the graphene sheets.^51^ Also, an increase in *E*_gap_ from 1.896 eV (CdO) to 2.182 eV (graphene–CdO complex) has been recorded due to the interaction. Such an effect suggests that the formed G–CdO complex is more stable than isolated CdO nanoparticles.^52^ The calculated dipole moment (DM) at the same level of theory for the G–CdO composite was found to be 3.97 Debye compared to 0 Debye for single G and CdO systems. The dipole moment is an important electronic property that measures the charge distribution asymmetry and the intermolecular interactions inside the molecule. The difference in electronegativity between Cd atom (1.69) and C atom (2.55) leads to induce a charge transfer from the Cd atom to the sheet, which in turn induces the dipole moment. Accordingly, the energy change in the electron transfer of the G–CdO composite decreased to −8.516 e. V, which indicates that the charge transfer interaction is energetically favorable (as Δ*E* < 0). In the same context, the value of Δ*N*_max_ (=3.623 eV) emphasizes the presence of intramolecular charge transfer within the composite, which elucidates its reactivity. When the ratio (Δ*E*/Δ*N*_max_) = 0 this means the compound is saturated with electrons and no charge transfer occurs. The calculated total electron energy of the ground state for the G–CdO complex is −31 789.8 eV showing minimum total energy among all three systems (−25082.1 eV for graphene and −6707.2 eV for CdO).

**Table 5:** Energy gap, HOMO, LUMO, potential (*μ*), hardness (*η*), electronegativity (*χ*), index of electrophilicity (*ω*), index of softness (*σ*), maximal charge acceptance (Δ*N*_max_) and the energy change (Δ*E*) values (in eV) of graphene, CdO and their resultant complex estimated at the DFT, B3LYP with LANL2DZ basis set

System	*E*_HOMO_ (eV)	*E*_LUMO_ (eV)	*E*_gap_ (eV)	*μ* (eV)	*η* (eV)	*χ* (eV)	*ω* (eV)	*σ* (eV)	Δ*N*_max_ (eV)	Δ*E* (eV)	Total energy (eV)
G–CdO	−5.042	−2.861	2.182	−3.952	1.091	3.952	8.516	0.917	3.623	−8.516	−31789.8
CdO	−5.625	−3.729	1.896	−4.677	0.948	4.677	10.371	1.055	4.934	−10.371	−6707.2
G	−5.653	−1.627	4.026	−3.640	2.013	3.640	13.335	0.497	1.808	−13.335	−25082.1

As listed in Table 5, G–CdO has an energetic gap lower than that of plain G, which allows G–CdO to be more polarizable and chemically reactive. The potential ionization *I*, and affinity properties *A* are so important to calculate the absolute hardness *η* and the electronegativity *χ*. The CdO has the largest absolute affinity value, so that will be a better electron acceptor during the interaction with plain graphene, which will be the electron donor. G–CdO has the lowest potential ionization value of 5.042 eV, so it is a better Lewis base (electron donor). Thus, according to DFT calculations, G–CdO is predicted to have a % removal efficiency for lead ions higher than individual graphene and CdO. The global electrophilicity index (*ω*) is a measure of energy lowering during accepting electrons. The electrophilicity value of G–CdO was the lowest, which strengthens its ability to act as an electrophile compared to single graphene and CdO. Therefore, the G–CdO composite is speculated to bind with lead ions and act as a nucleophile more than plain graphene and CdO systems.

### Total and partial density of states (TDOS and PDOS)

**Fig. 4:**
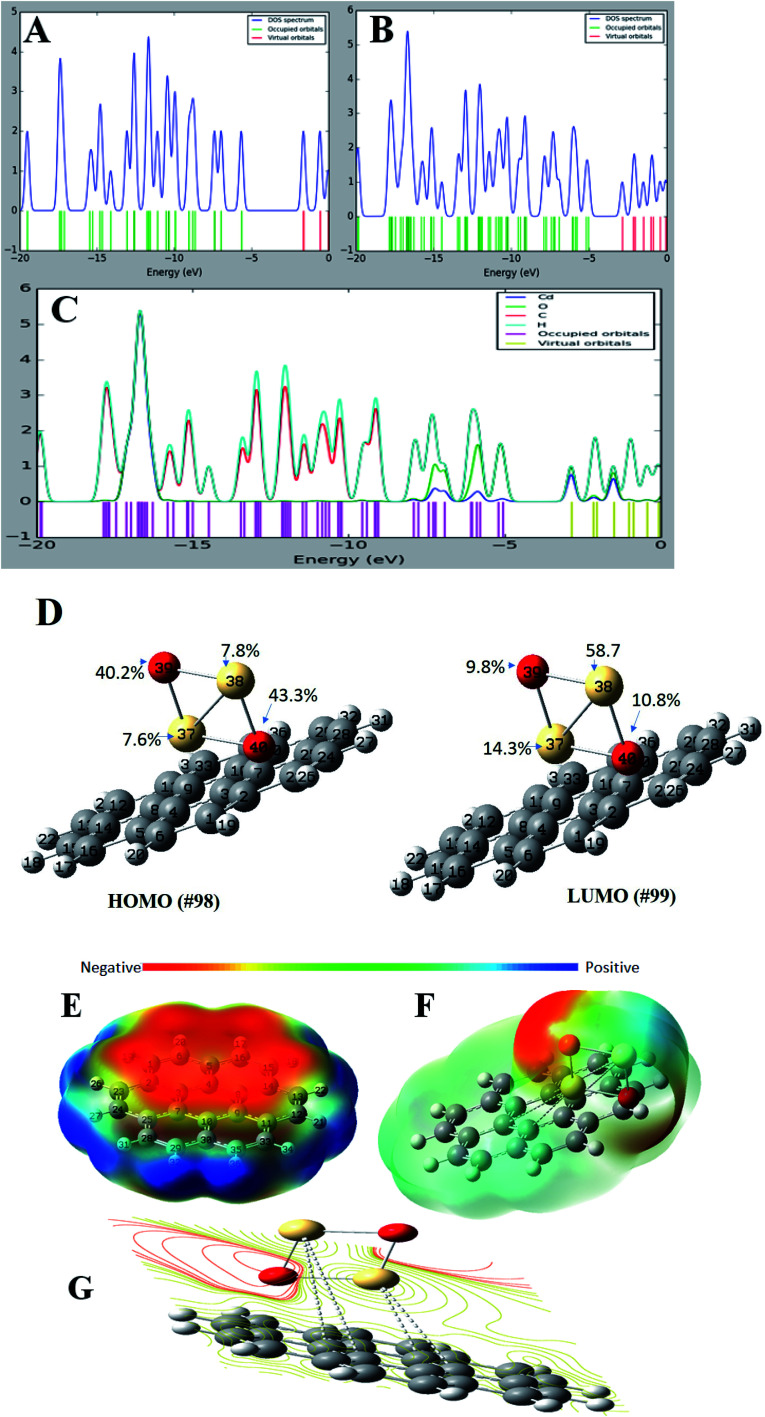
The total density of state (TDOS) of graphene (A), graphene–CdO (B), the partial density of state (PDOS) of graphene–CdO (C) and %orbital contribution for HOMO and LUMO, electrostatic potential maps for graphene (D), G–CdO composite (E) and electrostatic potential contour map of G–CdO (F and G), estimated at the DFT, B3LYP with LANL2DZ basis set.

For a better understanding of the deposition of CdO onto graphene sheets and its effect on the electronic properties of the formed composite, the total and partial density of states plots of the G–CdO complex was calculated and analyzed. The energy gap and energy level for both graphene and graphene–CdO molecules have been demonstrated in Fig. 4A–C. From the total density of states (TDOS) of G–CdO compared to the TDOS plot of single graphene, it is confirmed that the deposition of CdO on graphene sheets causes a significant shift in occupied and virtual orbitals. The electronic bands in the nanocomposite case, are continuous and levels of energy are increasing, while the energy gap is decreasing compared to that of individual graphene systems. The G–CdO has a small energy gap of 2.182 eV attributed to the chemical reactivity of the formed complex, while graphene recorded a high energy gap of 4.026 eV. Such finding explains the experimental results, whereas the lead removal efficiency of G–CdO composite was higher than that reported for plain graphene. For the graphene TDOS plot, both HOMO and LUMO are almost spread over the entire molecule. HOMO and LUMO of G–CdO are mainly localized on O and Cd atoms as demonstrated in PDOS, Fig. 4C. On the other hand, the LUMO+1 is spread over the complex while HOMO−1 is still localized on O and Cd atoms. The % orbital composition analysis of HOMO and LUMO according to the Beke method was performed using Multiwfn software^53^ and demonstrated in Fig. 4D. The density of HOMO is mainly composed of oxygen atoms, which indicates that the electrophilic attack can occur mostly at these sites. Meanwhile, LUMO density is dominated by sharing Cd atoms, thus nucleophilic attack is likely to occur at these sites.

### Molecular electrostatic potential (MEP)

It initially used to predict relative reactivity. It is the total interaction of the net charge distribution (electrons + nuclei) with partial charges, dipole moments, reactivity sites and electronegativity of a compound. Also, MEP explains the relationship between the structure and reactivity in molecule.^54^ The MEP plots at G and G–CdO surfaces are represented in Fig. 4E–G by a color range from red to blue color. The red color regions show the highly negative electrostatic potential (electrophilic sites) and the blue regions corresponding to a highly positive potential (nucleophilic sites). The area of zero potential is represented by green color. From Fig. 4E, an electrophilic attack will direct to pi graphene system, the region of red color and show high negative potential. The positive electrostatic potential, the region of blue color, is spread over the terminal hydrogen atoms of graphene sheets. The MEP mapped on the isodensity G–CdO surface, Fig. 4F, demonstrated a red negative area spread onto oxygen atoms of CdO NPs anchored on the graphene sheets, which suggests that electrophile ions such as Pb(ii) would preferentially attack G–CdO at oxygen positions. The yellowish color near localized at the pi system of graphene represents the area of less electrophilicity of the compound as compared to oxygen atoms, while the blue regions of the composite are spread on the cadmium atoms. The MEP contour map of G–CdO confirmed that oxygen atoms are electrophilic sites to remove cationic Pb(ii) ions, Fig. 4G.

### NBO analysis

Natural bond orbital (NBO) analysis is an efficient way to explore inter-and intramolecular bonding within the molecule such as charge transfer or conjugative process, charge distribution and donor–acceptor interactions. This analysis investigates the relationship between the filled NBOs (donors) and virtual or empty NBOs (acceptors). Such interaction is proportional to the stabilization energy *E*^2^ resulting from the second-order Fock-matrix analysis. Therefore, the greater extent of donor–acceptor interaction and conjugation the greater is the values of *E*^2^ and the charge transfer between the orbitals (filled and empty) of the whole system. Such interaction leads to a decrease in the occupancy from the idealized Lewis structure (lone pair or bonding) to a non-Lewis virtual orbital (anti-bonding or Rydberg). The most important interactions between Lewis type and non-Lewis type NBO along with, *E*^2^, their stabilization energies are calculated and listed in Table S1.† The perturbation theory analysis of the Fock matrix depicts strong intramolecular interactions, these various kinds of donor Lewis–acceptor non-Lewis interactions play a great role in the composite stabilization. The intramolecular interactions occur by overlapping between BD*(C–C) and LP(1)*C to BD*(C–C) structure resulting in charge transfer, which leads to stabilization of the formed nanocomposite. The highest contributions of intramolecular charge transfer within the graphene sheet are from antibonding BD*(2) C30–C35, BD*(2) C11–C12 to BD*(2) C9–C10, from BD*(2) C2–C3 to BD*(2) C23–C24 and from BD*(2) C5 – C6 to BD*(2) C15–C16 with stabilization energy of 309.27, 262.6, 201.38 and 112.89 kJ mol^−1^, respectively. Other significant intramolecular interactions are from LP*(6)Cd38 to LP*(8)Cd37 corresponds to stabilization energy 87.7 kJ mol^−1^. The charge transfer from LP*(1) C4 to BD*(2) C5–C6, from LP (1) C13 to BD*(2) C11–C12 and from LP*(1) C33 to BD*(2) C30–C35 corresponds to 84.64, 82.26 and 82.16 kJ mol^−1^, respectively. Such results match well with those previously conducted after optimization and correlate with the fatal function of density donation during the anchoring of CdO onto graphene sheets. However, the graphene system could also accept electronic sharing from Cd atoms *via* the back-donation process besides its tendency to give electrons.

### RDG and ELF analyses

**Fig. 5:**
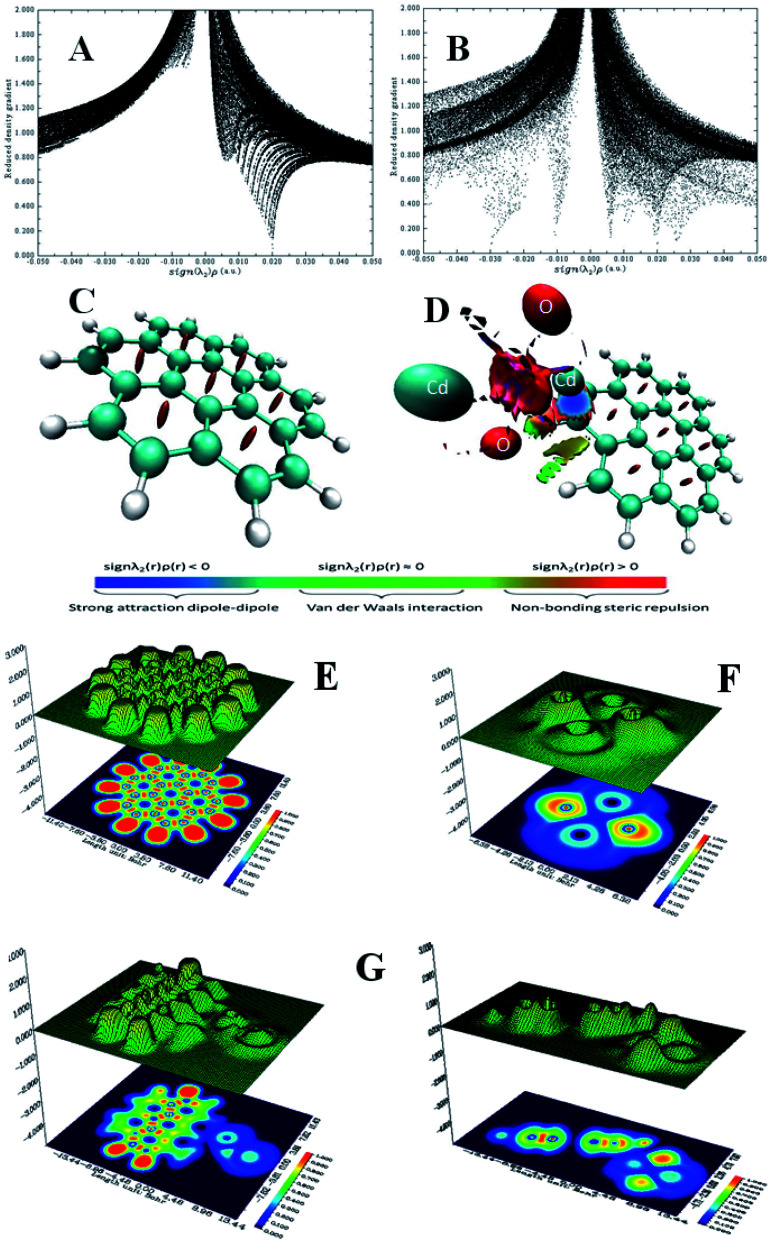
RDG scatter graph of G (A), G–Cd (B) and RDG color isosurface of G (C), G–Cd (D), ELF shaded surface map with projection for G (E), CdO (F) and G–Cd (G).

The RDG analysis is a non-covalent intermolecular identification and visualization tool. It is a dimensionless function based on the density *ρ*(*r*) and its derivatives to analyze the various kinds of intermolecular forces and can be expressed as:
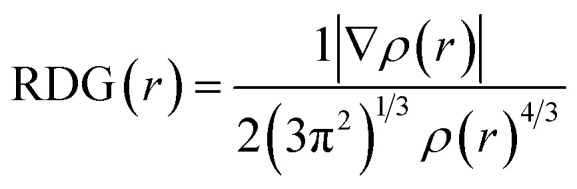


There is a direct correlation between the magnitude of the intermolecular interactions and both second derivative eigenvalue (*λ*_2_) of the Hessian matrix and *ρ*(*r*) in each point of the isosurface as it equals to the product of *ρ* and signs of *λ*_2_.^55^ Each specific value of RDG on the *Y*-axis has a region on the *X*-axis that can be classified into three types, strong attraction corresponds to sign *λ*_2_(*r*)*ρ*(*r*) < 0, strong repulsion corresponds to sign *λ*_2_(*r*)*ρ*(*r*) > 0 and weak interaction corresponds to sign *λ*_2_(*r*)*ρ*(*r*) ≈ 0. Therefore, to study the non-covalent reaction within the G–CdO composite, the RDG(*r*) *vs.* sign(*λ*_2_)*ρ*(*r*) function plot (scatter graph) is investigated at low gradient regions and is compared to the G scatter graph, Fig. 5A and B. Applying 0.5 as an RDG isosurface reference value, the scatter graph of plain graphene, Fig. 5A, characterizes spots at the area with sign *λ*_2_(*r*)*ρ*(*r*) > 0 due to the steric clash in the ring structures. The RDG scatter graph of G–CdO, Fig. 5B, reveals that the weak non-covalent interactions are predominant forces in the G–CdO complex as the spots at the region around zero. Further, the G–CdO graph shows a spike at sign *λ*_2_(*r*)*ρ*(*r*) > 0 indicating a steric interaction inside the graphene rings, as elucidated in the G scatter graph. In addition, the spike at negative density values indicates the attractive stabilizing dipole–dipole interaction due to the binding of CdO onto the G surface. To visualize the non-covalent interaction locations, Fig. 5C and D show the colored low reduced density gradient isosurfaces based on the sign *λ*_2_(*r*)*ρ*(*r*) value. The red regions show destabilizing repulsive steric nonbonding positive clashes while the blue region depicts the negative attractive dipole–dipole forces. The green region with values close to zero indicates weak dispersion-stabilizing interactions such as π stacking and van der Waals interactions. The obvious significant changes in the G isosurface features after the interaction with CdO indicate that the graphene/CdO interactions were strong and the system displays strong van der Waals interactions. Therefore, the RDG analysis is in good agreement with that conducted with NBO analysis.

ELF depends on the kinetic energy density and it is an important tool to visualize the electron pair location and size, which is interpreted to describe the chemical bonding. The ELF values take the numbers from 0 and 1, where zero value indicates no electron. Low values (<0.5) mean regions with delocalized electrons, while high ELF values (>0.5) describe domains with localized bonding and antibonding. Domains with perfect localization take the value of 1. Fig. 5E–G shows the projection map with the distribution of ELF for the studied nanomaterials. In Fig. 5E, single graphene shows no lone pair electrons while CdO in Fig. 5F shows localization around oxygen atoms. A significant change has been observed for graphene ELF map after anchoring of CdO at the surface of graphene, Fig. 5G. The side view of G–CdO clearly shows that lone-pair electrons are localized around oxygen atoms after doping graphene with CdO NPs as the ELF values are around 0.8–0.9.

### Repeatability and regeneration efficiency

**Fig. 6:**
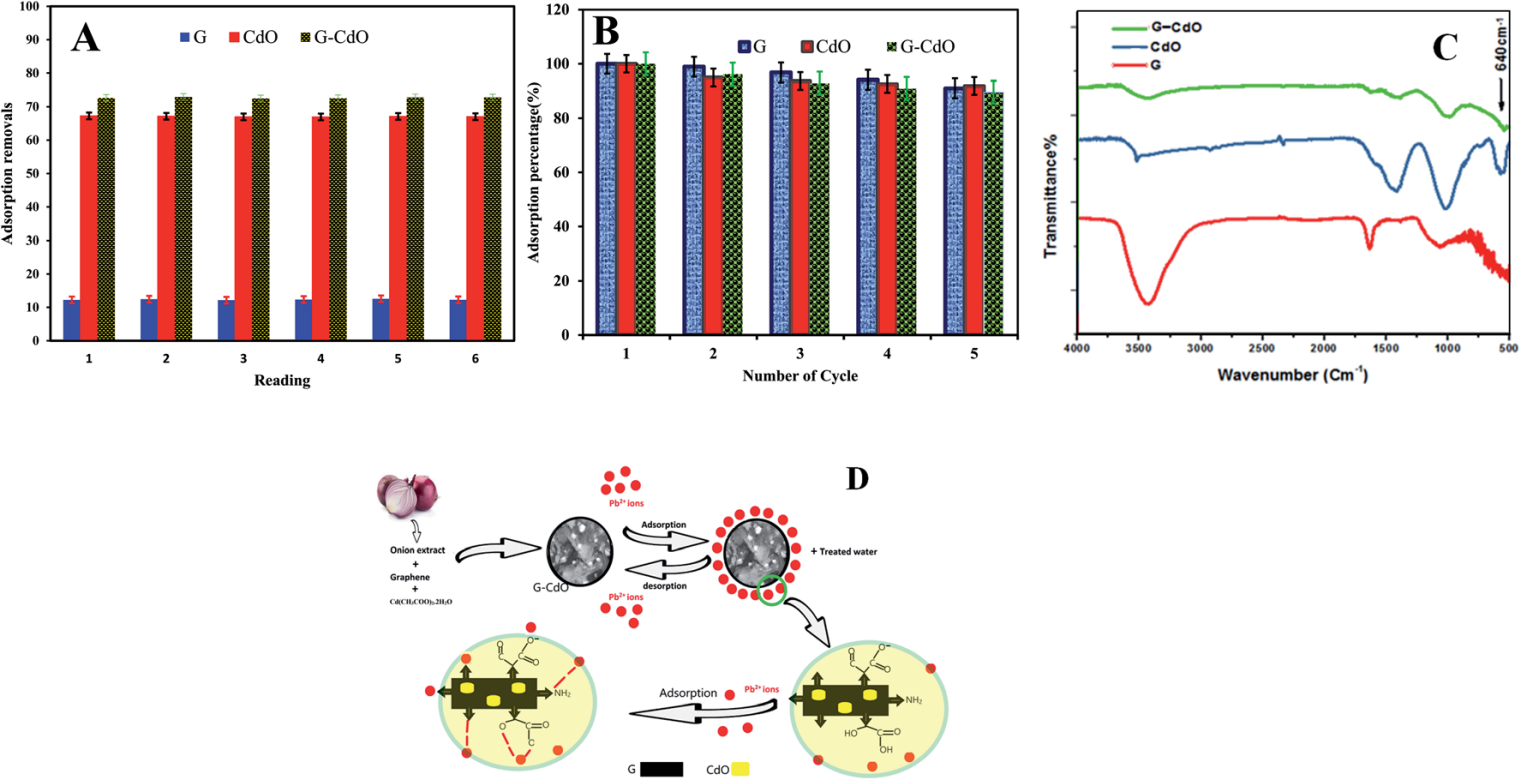
Removal of lead ions by graphene, CdO and G–CdO nano adsorbents: (A) intra-day repeatability for *n* = 8 and (B) regeneration efficiency for four consecutive cycles, (C) FTIR spectra after adsorption process, (D) the adsorption mechanism.

The intra-day repeatability (*n* = 8) was performed to evaluate the precision of removal. The precision expressed as percentage relative standard deviations (% RSDs) was 1%, Fig. 6A. The results obtained confirmed that the prepared nanoadsorbents can be suitable adsorbents for the preconcentration of lead in aqueous samples.

The two most pivotal features, which a good adsorbent should show are reusability and durability. To assess the possibility of regeneration and reusability of prepared nanomaterials as adsorbents, desorption experiments were carried out with 0.1 N HCl as an eluent and pH kept >2 to prevent leaching of CdO out of graphene. After extraction in an acidic medium, the recycled G–CdO was washed and reused again. The impact of four consecutive adsorption–desorption cycles was studied, Fig. 6B. The sorption efficiency of prepared nanomaterials towards lead decreases with the rise in the number of regeneration cycles. Such a decrease may be assigned to the inefficient removal of chemisorbed lead ions from the nanoadsorbent material, although no leaching of Cd^2+^ in the solution of HCl was found. The analysis confirmed that the rate of Pb(ii) metal is still more than 89.6% after repeated four cycles, indicating that the prepared nanomaterials have good recyclability as adsorbents. Thus, the surface of the nanoadsorbent under investigation gained positive charges and Pb(ii) ions almost desorbed into the acidic solution, indicating that the mechanism of ion exchange may be included in the sorption reaction. It can be concluded that, significantly, graphene-based CdO nanocomposite shows reusability and durability properties even after 4 regenerations. The marked regenerability of G–CdO can be assigned to its stable structure, facilitating its application in environmental treatment as no cadmium was detected in the solution, demonstrating that the nanocomposite was stable under the employed experimental conditions.

### Adsorption mechanism

The mechanism of lead uptake onto nano G–Cd composite is based on the adsorption isotherm and kinetics data discussed previously, as well as experimental FTIR data shown before and after the lead adsorption process.

G–CdO and CdO were successfully prepared using reducing and stabilizing agents present in the onion extract. FTIR analysis of the onion extract and nanomaterials is similar to some shifts due to the capping of nanoparticles with onion compounds. Thus, CdO NPs induced the coating of graphene sheets with many organic moieties. Also, FTIR confirmed the covalent bonding between graphene sheets and the modified CdO surface, while the SEM/TEM images showed the CdO NPs anchored at the surface of graphene sheets.

To study the mechanisms of as-prepared nanoadsorbents for Pb removal, an FTIR study was carried out. Comparing the FT-IR analysis before and after adsorption, it was found that the intensities of the peaks at around 3440 cm^−1^ (stretching vibration of H-bonded and N–H bond) and 1634 cm^−1^ (carboxylic) decreased and shifted after lead adsorption, Fig. 6C. Newly formed Pb–O stretching at 665 cm^−1^ appeared.^56^ The above analysis revealed the reduction of N–H and H-bonds in the functional groups and the replacement of lead ions in these active sites during the adsorption process. Similar results have been reported^47^ and confirmed a mechanism based on the formation of complexes or ion exchange with organic groups that wrapped nanomaterials. In the other work, Zhang *et al.*^57^ confirmed that both amine and carbonyl/hydroxyl groups within N doped adsorbent are responsible for lead adsorption. Li *et al.*^57^ used a polydopamine and MnO_2_ coating to remove Pb(ii) ions and they observed that the N contained groups in polydopamine also bind with lead ions. Besides, they have conducted that both amine and carbonyl/hydroxyl groups contribute to lead uptake through chelation. Fig. 6D demonstrates the suggested mechanism for lead removal by G–CdO nanocomposite. Therefore, the high removal capacity of G–CdO is related to lead chelation onto the bioactive organic layer located on CdO, and sorptive sites on graphene sheets as well as oxygen atoms of CdO as discussed in DFT calculations. Thus, G–CdO nanocomposite is highlighted as having outstanding adsorption performance towards Pb(ii) from the environment.

## Conclusions

CdO NPs and G–CdO composite were prepared and characterized. Results of FT-IR, SEM, EDX, TEM, XRD, UV-Vis analyses demonstrated that CdO NPs and G–CdO composites were successfully synthesized. Taken together, the integration of graphene and CdO NPs created a novel nanomaterial that potentially removes lead from an aqueous solution. The results of the batch adsorption experiments implied that the maximum adsorption capacities of CdO and G–CdO were 398 and 427 mg g^−1^, respectively. Pseudo-second order kinetic model and Langmuir isotherm explained the process. The thermodynamic studies were conducted through spontaneous, physical, and endothermic sorption reactions. The experimental results of %lead removal matched well with the DFT theoretical study of the nanocomposite tested. The energy of the perturbation of the Fock matrix depicted strong intramolecular interactions. Various kinds of donor Lewis–acceptor and non-Lewis interactions play a great function in composite stabilization. The RDG analysis is in good agreement with that conducted with NBO analysis as a significant change in the G–CdO isosurface features after the interaction with CdO indicates that graphene/CdO interactions were strong and the system displays strong van der Waals interactions. Also, the study confirmed that the uptake of lead ions onto prepared nanomaterials was mainly attributed to the electrostatic attraction forces and surface complexation. Additionally, CdO NPs and G–CdO composites could be regenerated and used repeatedly at least four times without significant loss in performance, allowing the as-prepared nanoadsorbents to be potentially applied to *in situ* lead removal from aqueous solutions.

## Conflicts of interest

There are no conflicts to declare.

## Supplementary Material

RA-011-D1RA04754J-s001
