# Supplementary material for: Cadmium oxide nanoparticles/graphene composite: synthesis, theoretical insights into reactivity and adsorption study
Source: RSC Adv. 2021 Aug 9;11(43):27027–41. doi: 10.1039/d1ra04754j (PMC9037664; doi:10.1039/d1ra04754j)
Supplement: RA-011-D1RA04754J-s001 [file RA-011-D1RA04754J-s001.pdf]

Electronic Supplementary Material (ESI) for RSC Advances.
This journal is © The Royal Society of Chemistry 2021

Supporting Information

Cadmium oxide nanoparticles/graphene composite: Synthesis, theoretical insights into reactivity and adsorption study

Dalal Z. Husein[(1),], Reda Hassanien[(1)], Mona Khamis[(1)]*

[(1)]*Chemistry Department, Faculty of Science, New Valley University, El-Kharja72511, Egypt*

**E-mail: dalal_husein@yahoo.com, dalal_husein@scinv.au.edu.eg*

Contents

- **The calculated values of stabilization energies, E^2, and its related interactions of Lewis and non-Lewis orbitals.**

Table S1 The calculated values of stabilization energies, E^2, and its related interactions of Lewis and non-Lewis orbitals.

Donor NBO (i)	Acceptor NBO (j)	E^2 kcal/mol	a.u.	a.u.
BD (2) C 2 - C 3	LP (1) C 1	62.47	0.1	0.086
	LP*(1) C 4	51.15	0.14	0.089
	BD*(2) C 7 - C 25	19.27	0.29	0.067
	BD*(2) C 23 - C 24	15.95	0.29	0.065
BD (2) C 5 - C 6	LP (1) C 1	71.95	0.11	0.094
	LP*(1) C 4	53.61	0.15	0.092
	BD*(2) C 15 - C 16	18.54	0.29	0.069
BD (2) C 7 - C 25	BD*(2) C 2 - C 3	21.88	0.27	0.069
	BD*(2) C 9 - C 10	22.23	0.28	0.071
	BD*(2) C 23 - C 24	15.75	0.28	0.063
	BD*(2) C 28 - C 29	16.39	0.28	0.064
BD (2) C 8 - C 14	LP*(1) C 4	55.44	0.14	0.091
	LP (1) C 13	51.89	0.13	0.086
	BD*(2) C 9 - C 10	21.81	0.28	0.071

	BD*(2) C 15 - C 16	16.74	0.28	0.064
BD (2) C 9 - C 10	BD*(2) C 7 - C 25	22.18	0.28	0.071
	BD*(2) C 8 - C 14	22.48	0.27	0.071
	BD*(2) C 11 - C 12	23.37	0.26	0.071
	BD*(2) C 30 - C 35	22.05	0.27	0.069
BD (2) C 11 - C 12	LP (1) C 13	69.44	0.13	0.097
	LP*(1) C 33	56.94	0.13	0.089
	BD*(2) C 9 - C 10	22.7	0.28	0.071
BD (2) C 15 - C 16	BD*(2) C 5 - C 6	18.58	0.27	0.068
	BD*(2) C 8 - C 14	16.37	0.3	0.066
BD (2) C 23 - C 24	BD*(2) C 2 - C 3	17.2	0.28	0.065
	BD*(2) C 7 - C 25	17.61	0.29	0.067
BD (2) C 28 - C 29	BD*(2) C 7 - C 25	16.6	0.3	0.066
	BD*(2) C 30 - C 35	18.01	0.29	0.068
BD (2) C 30 - C 35	LP*(1) C 33	69.49	0.13	0.097
	BD*(2) C 9 - C 10	21.32	0.28	0.069
	BD*(2) C 28 - C 29	19.04	0.28	0.069
LP (1) C 1	BD*(2) C 2 - C 3	51.43	0.18	0.098
	BD*(2) C 5 - C 6	73.15	0.16	0.11
LP*(1) C 4	BD*(2) C 2 - C 3	72.96	0.14	0.106
	BD*(2) C 5 - C 6	84.64	0.12	0.105
	BD*(2) C 8 - C 14	69.2	0.14	0.105
LP (1) C 13	BD*(2) C 8 - C 14	62.22	0.15	0.102
	BD*(2) C 11 - C 12	82.26	0.14	0.112
LP*(1) C 33	BD*(2) C 11 - C 12	65.27	0.14	0.099
	BD*(2) C 30 - C 35	82.16	0.14	0.112
BD*(2) C 2 - C 3	BD*(2) C 23 - C 24	201.38	0.01	0.077
BD*(2) C 5 - C 6	BD*(2) C 15 - C 16	112.89	0.02	0.072
BD*(2) C 11 - C 12	BD*(2) C 9 - C 10	262.6	0.01	0.079
BD*(2) C 30 - C 35	BD*(2) C 9 - C 10	309.27	0.01	0.082
LP*(6)Cd 37	LP*(8)Cd 37	48.58	0.32	0.207
	LP*(9)Cd 37	9.97	0.21	0.078
	RY*(6)Cd 37	19.77	3.4	0.473
	RY*(9)Cd 37	13.59	1.2	0.233
	LP*(9)Cd 38	35.63	0.64	0.261
LP*(7)Cd 37	LP*(7)Cd 38	9.46	0.01	0.028
LP*(6)Cd 38	LP*(8)Cd 37	87.7	0.31	0.251
	LP*(9)Cd 37	42.86	0.2	0.145
	LP*(8)Cd 38	10.49	0.1	0.05
	LP*(9)Cd 38	76.86	0.63	0.346
	RY*(4)Cd 38	31.74	2.59	0.472
	RY*(6)Cd 38	25.47	1.53	0.325

	RY*(7)Cd 38	27.3	0.58	0.208
	RY*(9)Cd 38	22.5	0.39	0.154
	RY*(3) O 39	9.99	1.17	0.178
LP (1) O 39	LP*(9)Cd 37	15.18	1.01	0.112
LP (2) O 39	LP*(7)Cd 37	9.33	0.23	0.041
LP (3) O 39	LP*(6)Cd 37	33.33	0.33	0.099
LP (4) O 39	LP*(6)Cd 37	29.74	0.22	0.074
LP (3) O 39	LP*(6)Cd 38	12.24	0.34	0.063
LP (4) O 39	LP*(6)Cd 38	40.47	0.23	0.091
	LP*(8)Cd 38	12.61	0.33	0.062
LP (3) O 40	LP*(6)Cd 37	34.03	0.31	0.097
	LP*(8)Cd 37	14.43	0.62	0.088
LP (4) O 40	LP*(6)Cd 38	63.17	0.25	0.119
